# Metasurface for highly-efficient on-chip classical and quantum all-optical modulation

**DOI:** 10.1038/s41377-022-00934-1

**Published:** 2022-07-28

**Authors:** Siqi Yan, Jianji Dong

**Affiliations:** Wuhan National Laboratory for Optoelectronics, School of Optical and Electronic Information, Huazhong University of Science and Technology, Wuhan, China

**Keywords:** Optical properties and devices, Metamaterials

## Abstract

Metasurface made of artificially two-dimensional structured subwavelength-scaled nanostructures gives rise to unprecedented efficient way to realize on-chip all-optical modulation, in both classical regime and quantum regime.

As a unique concept in the area of photonics, metamaterials^1^ refer to the periodic subwavelength structures whose electro-magnetic properties could not be found in natural materials. Thanks to its remarkable electromagnetic features including negative-index^2^, zero-index^3^ and ultra-high-index materials^4^, metamaterials have long been a research focus in the past two decades and ground-breaking photonic phenomena have been demonstrated. However, the difficulties in the micro- and nano-fabrication processes of the three-dimensional (3D) structures of the metamaterials significantly hinder its potential practical applications.

When the 3D metamaterials degenerate to planar two-dimensional (2D) structures, the difficulty in the fabrication process is significantly reduced since it can readily utilize the existing fabrication process such as lithography, dry etch and nanoimprint. Therefore, the researches of metasurfaces become a rapidly growing field since it can provide degrees of freedom in both designing and inhomogeneity over a thin interface with a low fabrication complexity. Numerous meta-devices have been reported such as meta-lens^5^, vortex beam generator^6^, holography^7^ and so on.

Besides the typical applications of the metasurfaces mentioned above, new areas are found to be perfectly matched with metamaterials, such as the all-optical modulation. In the all-optical modulation scheme, a pump light is normally employed to manipulate the physical property of the signal light including intensity, phase or polarization. Compared to other modulation techniques, the all-optical modulation could reach the highest bandwidth up to THz. Therefore, it is widely applied in optical-interconnects, optical-computing and ultrafast molecular spectroscopy. However, up to now, most of the all-optical modulation schemes are limited in the visible or near-infrared wavelength range and the all-optical modulator for wavelengths larger than 6 μm remains challenging due to the inherent optical absorption as well as the weak nonlinearity of the optical materials. To address this issue, the metasurface becomes a perfect solution.

Now, writing in this issue of *Light: Science & Applications*, Yu Yao and his colleagues at the Arizona State University in USA combined the metallic metasurface with graphene to implement efficient and fast all-optical modulators above 6 μm^8^. It should be pointed out that although graphene could absorb mid-infrared light due to its gapless band structure, the atomic thickness of graphene results in the low light absorption within graphene and causes high pump power^9^. To address this issue, Yu Yao and co-authors designed a tunable graphene-metallic metasurface absorber (GMMA) to enhance the light-graphene interaction at both signal wavelength and pump wavelength. The proposed GMMA contained an Al back reflector, a Al_2_O_3_ spacer layer, a plasmonic metasurface and a graphene layer on top. The pump light experienced a one-order of magnitude higher absorption within graphene thanks to the plasmonic enhancement within the nanogaps between the coupled nano-antenna of the metasurface. As a result, an ultra-low pump fluence less than 70 μJ/cm^2^ was experimentally demonstrated. Moreover, taking advantage of the ultrafast photo-carrier relaxation times in graphene, the all-optical modulator also held an ultrafast response time of picosecond scale. One could expect the proposed structures to widely benefit the potential applications of the mid-infrared optical modulation such as remote sensing, biomedical diagnostics and astronomical applications (Fig. 1).

**Fig. 1:**
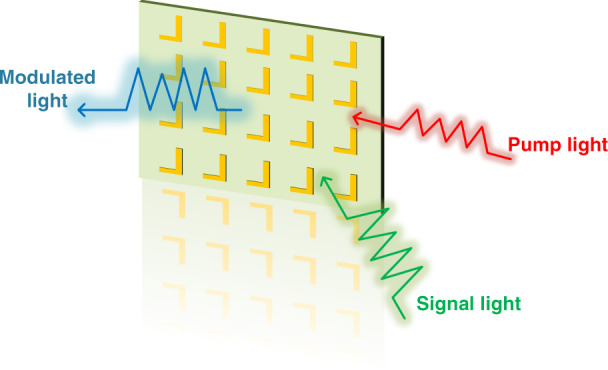
Operation principle of all-optical modulation. Schematic of the all-optical modulation based on the metasurface

Besides the all-optical modulation within the classical regime, the metasurface can also find its place in the quantum regime. The joint research team from Nankai University, University of Science and Technology of China, The University of Hong Kong and Shanxi University demonstrated a novel the quantum states all-optical manipulation method based on the nonlinear metasurface in this issue of *Light: Science & Applications*^10^. The metasurfaces were formed by an anisotropic structured nanostructure layer covered by a photoisomerizable ethyl red film. The polarization-entangled quantum states were therefore efficiently manipulated by optically switching the transmission contrast and phase retardation between the orthogonally polarized photons, thanks to the enhanced optical response of the metasurface. This impressive combination of metasurfaces and quantum regime could pave the way for the reconfigurable on-chip quantum systems in the future.

From classical regime to quantum regime, it is always a research focus of the scientific community to control light by another light. And metasurface plays important roles in both regimes to realize fast and efficient all-optical modulation.
